# Prediction and Characterization of Cationic Arginine-Rich Plant Antimicrobial Peptide SM-985 From Teosinte (*Zea mays* ssp. *mexicana*)

**DOI:** 10.3389/fmicb.2020.01353

**Published:** 2020-06-19

**Authors:** Abdelrahman M. Qutb, Feng Wei, Wubei Dong

**Affiliations:** ^1^Department of Plant Pathology, College of Plant Science and Technology and the Key Lab of Crop Disease Monitoring and Safety Control in Hubei Province, Huazhong Agricultural University, Wuhan, China; ^2^Department of Agricultural Botany, Faculty of Agriculture, Al-Azhar University, Cairo, Egypt; ^3^State Key Laboratory of Agricultural Microbiology, College of Life Science and Technology, Huazhong Agricultural University, Wuhan, China

**Keywords:** antimicrobial peptide, AMP prediction, bacterial pathogens, membrane damage, teosinte

## Abstract

Antimicrobial peptides (AMPs) are effective against different plant pathogens and newly considered as part of plant defense systems. From prokaryotes to eukaryotes, AMPs can exist in all forms of life. SM-985 is a cationic AMP (CAMP) isolated from the cDNA library of Mexican teosinte (*Zea mays* ssp. *mexicana*). A computational prediction server running with different algorithms was used to screen the teosinte cDNA library for AMPs, and the SM-985 peptide was predicted as an AMP with high probability prediction values. SM-985 is an arginine-rich peptide and composed of 21 amino acids (MW: 2671.06 Da). The physicochemical properties of SM-985 are very promising as an AMP, including the net charge (+8), hydrophobicity ratio of 23%, Boman index of 5.19 kcal/mol, and isoelectric point of 12.95. The SM-985 peptide has amphipathic α-helix conformations. The antimicrobial activity of SM-985 was confirmed against six bacterial plant pathogens, and the MIC of SM-985 against Gram-positive indicators was 8 μM, while the MIC of SM-985 against Gram-negative indicators was 4 μM. The SM-985 interacting with the bacterial membrane and this interaction were examined by treatment of the bacterial indicators with FITC-SM-985 peptide, which showed a high binding affinity of SM-985 to the bacterial membrane (whether Gram-positive or Gram-negative). Scanning electron microscopy (SEM) and transmission electron microscopy (TEM) images of the treated bacteria with SM-985 demonstrated cell membrane damage and cell lysis. *In vivo* antimicrobial activity was examined, and SM-985 prevented leaf spot disease infection caused by Pst DC3000 on *Solanum lycopersicum.* Moreover, SM-985 showed sensitivity to calcium chloride salt, which is a common feature of CAMPs.

## Introduction

Antimicrobial peptides (AMPs), defined as small-molecular-weight proteins, are regarded as one of the substitutes for traditional antibiotics since they are small molecules and often have a wide range of antimicrobial activity, and AMPs have always been acknowledged for their low relative cytotoxicity. The innate immune system of nearly all organisms produces AMPs, including bacteria (Hassan et al., 2012), animals (Hancock and Scott, 2000), and plants (Alland et al., 2005). AMPs represent the first line of defense against plant bacterial pathogens (Ebbensgaard et al., 2015). AMPs can be isolated from almost all plant parts (Nawrot et al., 2014). Teosinte includes a group of four annual *Zea* species (Galinat, 1969). Mexican teosinte *Zea mays* ssp. *mexicana* and maize are members of the same family and share the present accepted ancestor (Doebley, 1992). George Beadle was the first to suggest that teosinte is the wild ancestor of maize, and a few main genes chosen by the people of Mexico over the last 10,000 years may have domesticated teosinte to maize (Beadle, 1939, 1972). Breeding programs and germplasm classification reports have shown that, in general, cultivated plants have comparatively lower rates of resistance to abiotic and biotic stresses compared with their wild ancestors (Rosenthal and Dirzo, 1997). cDNA is a complementary DNA copy of mRNA that is generated by the enzyme reverse transcriptase. cDNA library construction is a powerful tool for determining cell- and tissue-specific gene expression. cDNA is prepared from mRNA, and it has no inverting sequences such as introns. Consequently, the DNA reflects both expressible RNA and gene products (proteins) (Ying, 2004). The large scale of cDNA library members, which can be thousands, makes the screening for AMPs quite challenging. However, in recent years, many studies have used the cDNA library method to clone AMPs (Ronèeviæ et al., 2018; Wu et al., 2019; Tavares et al., 2020) due to its advantages such as enhanced fragments of efficiently transcribed genes and cloned AMPs from cDNA library, which can be actively manufactured via bacterial expression systems (Sharma et al., 2014) due to the lack of introns that might pose a problem when the target is manufactured as a eukaryotic protein in bacteria. In our lab, we screened cDNA libraries for AMPs via expression systems (Kong et al., 2018; Wu et al., 2020), but this is also a time- and cost-consuming approach. *In silico* prediction is a time- and cost-effective approach for large-scale screening and detection of AMPs (Liu et al., 2017). The detection of AMPs from databases has drawn interest as a field of structural genomics and bioinformatics. Many techniques have been used to classify AMPs from databases, including local alignments, regular expression (REGEX), activity prediction by machine learning algorithms, as well as three-dimensional (3D) structure predictions (Porto et al., 2017). Well-constructed AMP databases provide good foundations for AMP prediction. Several prediction approaches have been suggested in recent decades (Porto et al., 2012), using several algorithms (Liu et al., 2017) centered on several parameters. For example, CAMPR3 is developing predictive methods for AMPs that rely on machine learning algorithms such as the random forest (RF), discriminant analysis (DA), and support vector machines (SVMs) (Thomas et al., 2010). APD3 offers useful information on the peptide discovery timeline, description, terminology, bibliography, statistics, and calculation methods. The APD allows the successful scanning, configuration, and prediction of AMPs (Zhou and Huang, 2015). The DBAASP developed a new simple algorithm of prediction based on the physicochemical characteristics in charge of the tendency of the peptide to interact with the anionic bacterial membrane, including hydrophobicity, amphiphilicity, and net charge (Vishnepolsky and Pirtskhalava, 2014). AMPs with a large amount of positively charged amino acids such as arginine have high net charge values and are called cationic AMPs (CAMPs). CAMPs have strong antimicrobial activity against bacterial pathogens, slow resistance formation, and fast action (Ciumac et al., 2019). The α-helical AMPs are potent agents in mediating plant defense due to the antibacterial effect (Montesinos, 2007; Keymanesh et al., 2009). Their primary mechanism is to destroy the outer and plasma pathogen membranes, prevent membrane invasion, or pore formation, leading to cell lysis (Holaskova et al., 2014). In addition, other antimicrobial mechanisms have been described to affect essential cellular processes involving DNA and protein synthesis, cell wall synthesis, protein folding, and enzymatic activity (Nicolas, 2009). Bacterial plant diseases are responsible for major losses of crops and agricultural goods, and their protection relies primarily on chemical pesticides (Agrios, 2004). Some of these bacterial diseases are bacterial wilt of tomato, bacterial canker of tomato, bacterial blight of rice, bacterial leaf streak, and leaf spot disease of tomato, and these diseases are caused by *Ralstonia solanacearum* (Murthy et al., 2019), *Clavibacter michiganensis* ssp. *michiganensis* (Tancos et al., 2013), *Xanthomonas oryzae* pv. *oryzae* (Sharma et al., 2017), *Xanthomonas campestris* pv. *holcicola* (Navi et al., 2002), and *Pseudomonas syringae* pv. *tomato* DC3000 (Xin and He, 2013), respectively. Several pesticides have been banned due to their negative influence on the environment. However, because of the lack of active compounds, certain plant diseases of economic significance have faced management difficulties.

This study aimed to isolate novel AMPs with effective antimicrobial activity against some plant bacterial pathogens (Gram-positive and Gram-negative) from the Mexican teosinte (*Z. mays* ssp. *mexicana*) and to investigate the mechanism underlying this antimicrobial activity.

## Materials and Methods

### cDNA Library Construction

*Zea mays* ssp. *mexicana* seeds were grown in peat moss pots in a growth chamber at 28^°^C and under 14-h light/10-h dark conditions. After 21 days of growth, teosinte leaves were inoculated with PDA disks of *Rhizoctonia solani* AG1-lA (maize leaf blight pathogen) that were 48 h old. The sampling was performed by collecting leaves samples at 10 different time points: 0, 6, 12, 24, 36, 48, 60, 72, 86, and 96 h after inoculation. The leaf samples were directly frozen in liquid nitrogen and stored in a -80^°^C freezer. cDNA library construction of teosinte (*Z. mays* ssp. *mexicana*) was performed according to general procedures: total RNA extraction, mRNA purification, cDNA synthesis, incorporation of cDNA into pBE-S vector, transformation into *Escherichia coli* HST08, and transformation into *Bacillus subtilis* SCK6 super competent cells. The complete steps of constructing the teosinte cDNA library are mentioned in the Supplementary Data, and adaptors used for construction of the cDNA library are mentioned in Supplementary Table S1. The total RNA, mRNA, and cDNA were visualized in a gel (Supplementary Figure S1). Random colonies were chosen from the cDNA library, and colony PCR was conducted using vector pBE-S general primers (Supplementary Table S2). The colony PCR protocol was started at 95°C for 5 min, followed by 28 cycles were performed (95°C for 30 s, 56°C for 30 s, 72°C for 55 s), and then 72°C for 10 min. After the PCR protocol was finished, the quality of the teosinte cDNA library was checked by gel electrophoresis.

### cDNA Sequencing and Analysis

Colony PCR was performed for all recombinant colonies using pBE-S primers, and the colony PCR protocol was started at 95°C for 5 min, followed by 30 cycles were performed (95°C for 30 s, 56°C for 30 s, 72°C for 55 s), and then 72°C for 10 min. The band sizes were determined by gel electrophoresis. The PCR products were sent for sequencing (repeated three times) using the Sanger method (Sander et al., 1976) to determine the cDNA sequences, while the empty vector bands were eliminated. The Basic Local Alignment Search Tool (BLAST) was used at NCBI^1^, MM GDB^2^, and Maize GDB^3^ to confirm the sources of these cDNA sequences and their relationship to genus *Z. mays*. cDNA sequences were translated from nucleic acids to amino acid sequences using a translation server^4^ grounded on the codon sequence of each amino acid according to the NCBI codes (Osawa et al., 1992; Jukes and Osawa, 1993).

### Bioinformatics Analysis

#### *In silico* Prediction-Based Screening for AMPs

To screen the teosinte cDNA library, computational prediction tools were used. The selected amino acid sequences were uploaded in the FASTA format into AMP prediction servers. For general AMP prediction, different prediction servers were used, including CAMP_R__3_^5^ (Waghu et al., 2016), APD3^6^ (Wang, 2015), AMPA with threshold value 0.225^7^ (Torrent et al., 2012), DBAASP^8^ (Vishnepolsky and Pirtskhalava, 2014), and MLAMP^9^ (Lin and Xu, 2016). Specific AMP prediction servers have been used to narrow the spectrum of antimicrobial activities of the expected AMPs depending on the type of organism (anti-fungal, antibacterial or antiviral)^10^ (Joseph et al., 2012), iAMPpred^11^ (Meher et al., 2017), Antibp^12^ (Lata et al., 2007), and dbAMP^13^ (Jhong et al., 2019).

#### Peptide Characterization

The physicochemical properties of SM-985 peptide were predicted using two servers, APD3 (Wang, 2015) for the amino acid composition, hydrophobic ratio, Boman index, and molecular weight. Moreover, DBAASP (Vishnepolsky and Pirtskhalava, 2014) was used for the isoelectric point, net charge, and *in vitro* aggregation. The secondary structure of SM-985 was predicted using three different servers, PSIPRED^14^ (Buchan and Jones, 2019), I-TASSER^15^ (Roy et al., 2010), and PEPstrMOD^16^ (Singh et al., 2015). BLAST determined the similarity of SM-985 to other AMPs in five different AMPs databases: CAMP_R__3_ (Waghu et al., 2016), APD3 (Wang, 2015), DRAMP (Fan et al., 2016), MLAMP (Lin and Xu, 2016), and dbAMP (Jhong et al., 2019).

#### 3D Structure Prediction

The 3D structure of SM-985 was predicted by I-TASSER (Roy et al., 2010) and visualized using UCSF Chimera 1.14rc software. The 3D structure of SM-985 was validated by MolProbity (Williams et al., 2018) and ProSA-web (Wiederstein and Sippl, 2007). Conversely, the helical wheel diagram was designed by HeliQuest (Gautier et al., 2008).

#### Microbial Indicator Strains, Culture Media, and Bacterial Growth Conditions

In this study, the antimicrobial activity of SM-985 was screened against eight different bacterial indicators, such as the Gram-positive bacteria *Clavibacter fangii*, *C. michiganensis* ssp. *michiganesis*, and *B. subtilis* 168. Moreover, we used the Gram-negative bacteria *P. syringae* pv. *tomato* DC3000, *R. solanacearum*, *X. campestris* pv. *holcicola*, *X. oryzae* pv. *oryzae*, and *E. coli* BL21. Luria–Bertani (LB) medium was used to grow the genus *Xanthomonas*, King’s B (KB) medium was used to grow the genus *Pseudomonas*, and Nutrient agar (NA) medium was used to grow the remaining bacterial stains. Non-pathogenic bacterial indicators (*B*. *subtilis* 168 and *E. coli* BL21) were incubated at 37^°^C, while pathogenic bacterial indicators were incubated at 28^°^C.

#### SM-985 Peptide

SM-985 peptide was synthesized according to the Fmoc Solid Phase Peptide Synthesis (Fmoc SPPS) method at 97% purity, and peptide synthesis was performed by Genscript (United States) Co. Ltd. The company provided all the information on peptide characterization, including mass spectrometry and HPLC data.

#### Minimal Inhibition Concentration (MIC) and Minimal Bactericidal Concentration (MBC) Assays

The minimal inhibition concentration (MIC) and minimal bactericidal concentration (MBC) SM-985 peptide were determined against eight bacterial indicators, and these assays were carried out using the agar and broth dilution method (Wiegand et al., 2008) with some alterations. For each bacterial indicator, a single colony was gown in Mueller Hinton Broth (MHB) at 28 and 37^°^C for pathogenic and non-pathogenic bacterial indicators, respectively. The bacteria culture was diluted with MHB to reach a concentration of ∼1 × 10^6^ colony-forming units (CFU)/ml. A stock of 256 μM SM-985 in MHB was prepared, and in a microtiter plate, six serial dilutions of SM-985 peptide were prepared in the microtiter wells: 128, 64, 32, 16, 8, and 4 μM. Each well containing the peptide solution and the growth control well (without SM-985) were inoculated with the bacterial suspension, and the final concentration of the bacterial cells was ∼1 × 10^5^ CFU/ml. A sterile control was made without either bacterial or SM-985. The microtiter plate was incubated for 8 h at 28 and 37^°^C for pathogenic and non-pathogenic bacterial indicators, respectively. Serial dilutions were generated for the different SM-985 concentrations, growth control, and sterile control. The suitable dilution was plated on medium plates and incubated under the same conditions until colonies grew in the growth control. The MIC value was determined as the lowest SM-985 concentration that caused 80% inhibition of the growth control (Wu et al., 2014). The MBC value was calculated as the minimum SM-985 concentration that caused no bacterial growth (Kang et al., 2011).

#### Minimal Lethal Concentration (MLC) Assay

Bacterial suspensions (∼1 × 10^6^CFU/ml) were prepared using 10 mM phosphate buffer for each bacterial indicator (Van De Velde et al., 2010); the detailed steps of preparing the bacterial suspensions are mentioned in the Supplementary Methods. The bacterial suspension was treated with different final concentrations of SM-985 peptide, 128, 64, 32, 16, 8, 4, and 2 μM, in microtubes and labeled as treatments, while a microtube of the bacterial suspension was treated with dd water and labeled as a control. Both the treatments and control were incubated for 4 h (with gentle inversion each 1 h) at 28 and 37^°^C for pathogenic and non-pathogenic bacterial indicators, respectively. After incubation, serial dilutions were performed for both treatments and control. In medium plates in triplicate for both treatments and control, 100 μl of the suitable dilution (30–300 CFU per plate) was plated out, and then the plates were incubated until visible colonies grew at 28 and 37°C for pathogenic and non-pathogenic bacterial indicators, respectively. The MLC was determined as the minimum SM-985 concentration that caused no bacterial growth in the treated plates. This experiment was performed three times independently.

#### Cell Membrane Integrity Assay

Damage to the cytoplasmic membrane was assayed by propidium iodide (PI) uptake according to Van De Velde et al. (2010). For each bacterial indicator, a bacterial suspension ∼1 × 10^7^ CFU/ml was prepared using 10 mM phosphate buffer (pH 7.0). The bacterial suspension was treated with 10 μM SM-985 and labeled as the treatment. A microtube with a similar volume of the bacterial suspension was treated with dd water and labeled as the control. Both the treatment and control were incubated for 4 h (with gentle inversion for each 1 h) at 28 and 37^°^C for pathogenic and non-pathogenic bacterial indicators, respectively. After incubation, PI dye was added to both the treatment and control microtubes at 10 μg/ml and fixed under dark conditions for 15 min. The bacterial cells were washed out with a 10 mM phosphate buffer (pH 7.0) two times by centrifugation at 5000 r/min to remove PI residues, and then resuspended in the buffer. An Olympus BX61 laser scanning confocal microscope (Wang et al., 2017) and flow cytometry Cytoflex lx (Beckman Coulter, Brea, CA, United States) (Kwon et al., 2019) were used to visualize and measure the PI uptake, respectively. The wavelengths of the excitation and emission were 535 and 617 nm, respectively. The flow cytometer data were analyzed using CyExpert 2.4 software.

#### FITC-Labeled-SM-985 Peptide

FITC-labeled-SM-985 peptide was synthesized by Genscript (United States) Co. Ltd. The FITC-labeled SM-985 peptide was dissolved as described for the SM-985 peptide, considering the change in molecular weight, and used to evaluate the interaction between SM-985 and the cytoplasmic membrane of each bacterial indicator (Zhu et al., 2015a). Similar cell membrane integrity assay procedures were preformed, but 4 μM FITC-SM-985 was used instead of SM-985. After incubation with FITC-SM-985, the bacterial cells were washed with 10 mM phosphate buffer (pH 7.0) to remove the peptide residues and resuspended in the buffer. An Olympus BX61 laser scanning confocal microscope was used to visualize FITC fluorescence at wavelengths of 488 and 500–530 nm, respectively. A cell killing assay was carried out on *C. michiganesis* ssp. *michiganesis* and *P. syringae* pv. *tomato* DC3000 to investigate the influence of the FITC tag on SM-985 antimicrobial activity. Both bacterial indicator suspensions (∼1 × 10^6^ CFU/ml) were treated with 5 μM FITC-SM-985 for 4 h, while the control was treated with dd water. After incubation, serial dilutions were carried out for both the treatment and control. In medium plates in triplicate for both treatments and control, 100 μl of the suitable dilution (30–300 CFU per plate) was plated out, and then the plates were all incubated at 28^°^C until visible colony growth. Moreover, a cell membrane integrity assay was performed to verify the antimicrobial activity of 10 μM FITC-SM-985 peptide against seven bacterial indicators. The Leica TCS SP5 confocal microscope was used to determine both FTIC and PI fluorescence.

#### *In vivo* Antimicrobial Activity Assay

This experiment was carried out by a virulent bacterial strain of *P. syringae* pv. *tomato* DC3000 and two host *Nicotiana benthamiana* and *Solanum lycopersicum*. *N. benthamiana* plants grown in a growth room at 24^°^C (Yang L. Y. et al., 2018) (14/10 h, light/dark) for 5 weeks, while *S. lycopersicum* plants were grown at 28^°^C (14/10 h, light/dark) for 6 weeks. A bacterial suspension (∼1 × 10^6^ CFU/ml) of Pst DC3000 was prepared. The *in vivo* AMP was assayed on both hosts in two ways. The first was performed on both hosts. SM-985 peptide was added to Pst DC3000 at a final concentration 5 μM, while in the control tube, Pst DC3000 was treated with dd water. Both the treatment and control were incubated at 28^°^C for 4 h (with gentle inversion for each 1 h). After incubation, both *N. benthamiana* and *S. lycopersicum* plants were inoculated with Pst DC3000 (treatment/control) using the infiltration method (Van De Velde et al., 2010; Vandenbossche et al., 2013) on the abaxial surface. The hypersensitive reaction was determined on *N. benthamiana* after 2 days, while necrosis symptoms on *S. lycopersicum* were determined after 4 days. The second was only performed on *S. lycopersicum*, and the same steps were followed as described above; the incubation, however, was performed on the plant surface. Both the treatment and control were sprayed directly on both adaxial and abaxial surfaces of plant leaves. The leaf spot disease symptoms were determined 6 days later, and both experiments were performed in triplicate.

#### Scanning Electron Microscopy (SEM)

This experiment was performed on *C. michiganesis* ssp. *michiganesis* as a representative of other bacterial indicators. A bacterial suspension (∼1 × 10^7^ CFU/ml) was prepared and treated with 15 μM SM-985, while the control was treated with dd water; both treatment and control were incubated at 28^°^C (4 h with gentle inversion for each 1 h). Bacterial cells (treatment/control) were prepared to scanning electron microscopy (SEM) according to Wu et al. (2014) with some modifications, and they were handled in the same way. After incubation, the bacterial cells were collected and then fixed with 2.5% (v/v) of glutaraldehyde solution at room temperature for 2 h; then the solution of fixation was removed, and the bacterial cells were washed twice with 10 mM phosphate buffer (pH 7.0). The bacterial cells were dehydrated with 30, 50, 70, 90, and 100% ethanol solutions, respectively, and the bacterial pellet was dried under air for 20 min followed by a freeze dryer for 24 h until it achieved a powder form. Bacterial cells were lyophilized and coated with gold and then observed under a HITACHI SU8010 scanning electron microscope.

#### Transmission Electron Microscopy (TEM)

The transmission electron microscopy (TEM) assay was performed on *C. michiganesis* ssp. *michiganesis* as a representative of other bacterial indicators. The same steps as described for SEM were followed. However, after the incubation period, the bacterial cells were obtained and set to 2.5% (v/v) of the glutaraldehyde solution. The samples were then sent to the HZAU center of TEM (Wuhan, China) for preparation, and the bacterial cells were observed under a HITACHI H-7650 transmission electron microscope.

#### Influence of Calcium Chloride on SM-985 Antimicrobial Activity Assay

This assay was performed according to Van De Velde et al. (2010), with some modifications, on two bacterial indicators, Gram-positive bacteria *C. michiganesis* ssp. *michiganesis*, and Gram-negative bacteria *P. syringae* pv. *tomato* DC3000. For both bacterial indicators, a bacterial suspension (∼1 × 10^6^ CFU/ml) was prepared, and calcium chloride salt was added to each bacterial suspension at four different final concentrations: 0, 5, 10, and 20 mM. As a final concentration, 5 μM SM-985 was added to the bacterial suspensions with four calcium chloride concentrations, after which they were incubated at 28^°^C for 4 h. After incubation, serial dilutions were performed. In medium plates in triplicate for both the treatments and control, 100 μl of the suitable dilution to obtain 30–300 CFU per plate was plated out and incubated at 28^°^C until visible colonies grew. Finally, the influence of calcium chloride salt on SM-985 activity was measured by counting the CFU of each salt concentration. All these procedures in the whole experiment were performed in triplicate.

## Results

### Teosinte cDNA Library

The cDNA insertions of teosinte were cloned into *B. subtilis* SCK6, and the constructed cDNA library consisted of 2500 single colonies. Most of the randomly picked up colony band sizes were more than 500 bp (empty vector band size) due to cDNA insertions, which indicated the high quality of the cDNA library (Supplementary Figure S2). As a result of the colony PCR of the whole cDNA library, more than 2000 colonies had cDNA insertions with varied band sizes. The sequencing results revealed similarities among the 2000 insertions, and to avoid repetition, 500 colonies were eliminated. The cDNA insertions showed 100% similarity with genus *Zea* according to the BLAST results against NCBI, MM GDB, and Maize GDB.

### Bioinformatics Analysis

#### *In silico* AMP Prediction

**FIGURE 1:**
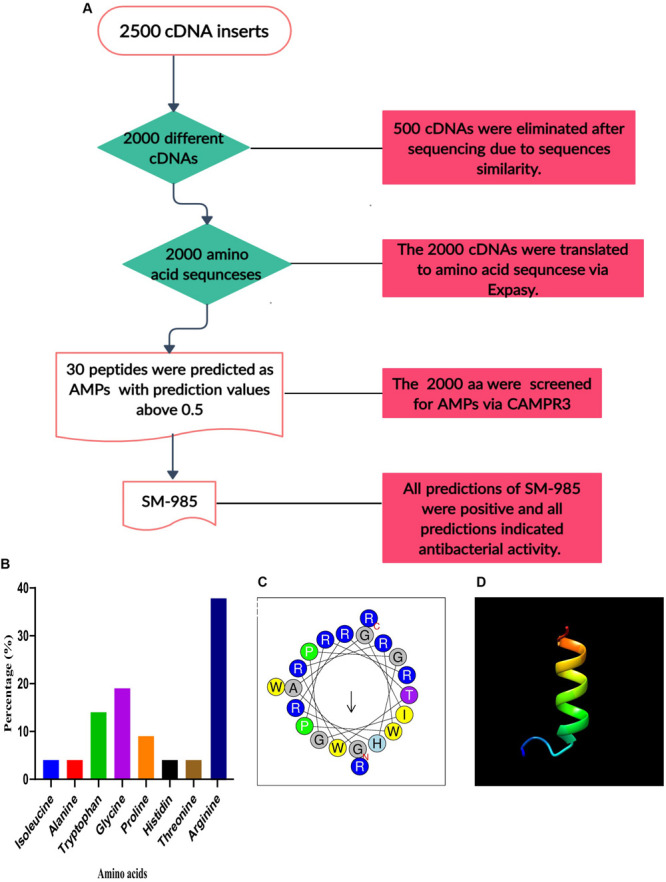
Bioinformatics analysis of SM-985. **(A)** Flow chart of peptide screening. **(B)** SM-985 composed of eight different amino acids, most with antimicrobial potentials, and rich in arginine (graph created with GraphPad Prism 8). **(C)** Helical wheel diagram of SM-985 peptide showing the amphipathic alpha-helix conformations. **(D)** The SM-985 predicted 3D structure contains an α-helix, as predicted by I-TASSER. The 3D structure model visualized using Chimera 1.14rc software. The 3D structure model of SM-985 was validated by ProSA-web software, the Z-score suggests that the peptide within a suitable area of structures. Regarding the MolProbity validation, the residues of SM-985 were within the favorable region.

After the translation of the 2000 cDNA inserts to amino acid sequences, each amino acid sequence was named based on size (the amino acid sequences were grouped under three groups of small sequences of 0–20 aa, medium sequences of 21–50 aa, and large sequences of greater than 50 aa), and the serial number of each sequence ranged from 1 to 2000. The 2000 sequences were screened for AMPs by the CAMPR3 prediction server (SVM algorithm), and based on the screening results, 30 sequences showed prediction values greater than 0.5 (Supplementary Table S3). SM-985 showed the height prediction value among the 30 sequences; the number of sequences filtered in each step is summarized in Figure 1A. Moreover, AMPA detected a stretch of essential antimicrobial amino acids from amino acid number 4 to number 20 within the SM-985 sequence, and this stretch had a propensity scale of 0.149 with 0% probability of misclassification. Generally, all predictions of SM-985 were positive (Supplementary Table S4) and all predictions indicated antibacterial activity (Supplementary Table S5).

#### Characteristics of SM-985 Peptide

Predicting SM-985 as AMP requires in-depth physicochemical sequence-based analysis. SM-985 is a small peptide with 21 amino acids; these 21 aa are composed of eight different amino acids as follows: arginine (R) 38%, glycine (G) 19%, tryptophan (T) 14%, proline (P) 9%, alanine (A) 4%, isoleucine (4%), histidine (4%), and threonine (4%). SM-985 is an arginine-rich peptide, according to APD3 (Figure 1B). The calculation of the physicochemical properties of SM-985 showed AMP properties due to the high net charge value (+8), high isoelectric point 12.95, hydrophobic ratio of 23%, protein-binding potential (Boman index) value of 5.19 kcal/mol, and *in vitro* aggregation value of zero. The secondary structure prediction by the servers PSIPRED, I-TASSER, and PEPstrMOD indicated that the SM-985 peptide contained an α-helix structure. The helical wheel diagram of SM-985 peptide had amphipathic α-helix conformations, in which the hydrophobic and the hydrophilic residues were placed on contradictory flanks of the α-helix (Figure 1C). The SM-985 peptide sequence showed no similarities to AMPs in the DRAMP, MLAMP, or dbAMP databases. Moreover, SM-985 had no significant similarities to either APD3 or CAMP_R__3__._ Thus, the BLAST results showed no similarity between the SM-985 peptide sequence and the other AMPs, confirming the novelty of the peptide.

#### The 3D Structure of SM-985

The α-helical 3D structure model was predicted by the I-TASSER server (Figure 1D). To prevent mistakes in selecting the correct peptide structural model, the ProSA-web and MolProbity methods can be used to refine and verify peptide models. The α-helical 3D structure of SM-985 was within the suitable quality range. The Z-score value obtained using the ProSA-web software, which suggests that the majority of data points in a multidimensional NMR range are not signal-occupied locations (Wiederstein and Sippl, 2007), showed that the peptides were within a suitable area of structures, indicating that they have characteristics of native structures (Würz and Güntert, 2017). Regarding the MolProbity validation, the residues of SM-985 were within the favorable region (right-handed α-helix), indicating high structural reliability. Conversely, the SM-985 peptide generated 94.7% of its residues in the favorable region and 5.3% in the permitted region.

#### Determination of MICs and MBCs of SM-985 Peptide

The MIC and MBC of SM-985 were investigated against all the bacterial indicators. The lowest SM-985 concentration causing 80% growth inhibition (MIC) of the Gram-positive bacteria *C. fangii*, *C. michiganesis* ssp. *michiganesis*, and *B. subtilis* 168 was 8 μM, while the lowest SM-985 concentration causing no bacterial growth (MBC) was 16 μM. The MIC values of SM-985 against the Gram-negative bacterial indicators *X. campestris* pv. *holcicola*, *X. oryzae* pv. *oryzae*, *P. syringae* pv. *tomato* DC3000, *R. solanacearum*, and *E. coli* BL21 were 4 μM, <4 μM, <4 μM, 4 μM, and 8 μM, respectively. The MBC values of SM-985 were 16 μM, 4 μM, 4 μM, 8 μM, and 16 μM, respectively (Table 1).

**TABLE 1:** The MIC and MBC values of SM-985 peptide against bacterial indicators.

Bacterial indicators	MIC (μM)	MBC (μM)
*C. fangii*	8	16
*C. michiganesis* ssp. *michiganesis*	8	16
*B. subtilis* 168	8	16
*X. campestris* pv. *holcicola*	4	16
*X. oryzae* pv. *oryzae*	<4	4
*P. syringae* pv. *tomato* DC3000	<4	4
*R. solanacearum*	4	8
*E. coli* BL21	8	16

*The MIC and MBC assays were performed according to the agar and broth method. (*<*) means no bacterial growth was noticed among all SM-985 concentrations.*

#### The MLC of SM-985 Peptide Causes Complete Death for All Bacterial Indicators

**FIGURE 2:**
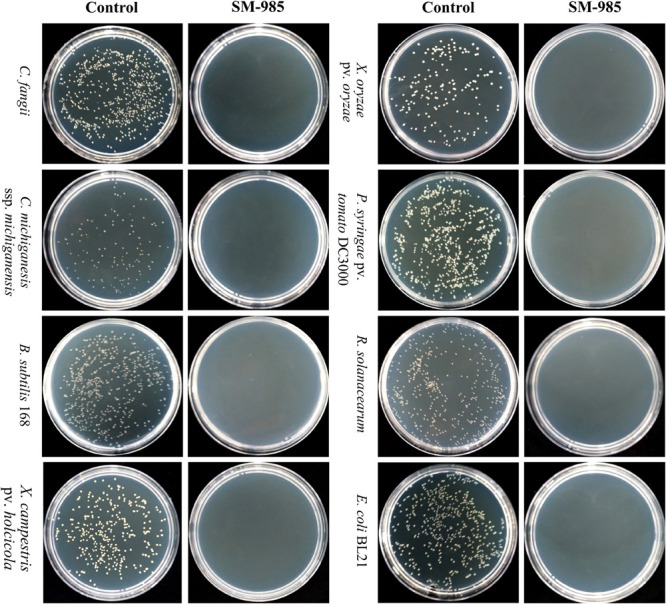
MLC of SM-985. No bacterial growth was noticed after all the bacterial indicators (Gram-negative and Gram-positive bacteria) were treated with SM-985 at ≤2 μM concentration for 4 h in phosphate buffer. However, the bacterial indicators grew well in the control treated with dd water.

This experiment was undertaken to assess the lowest concentration of SM-985 peptide, which causes complete death for all bacterial indicators, Gram-positive bacteria (*C. fangii*, *C. michiganesis* ssp. *michiganesis*, and *B. subtilis* 168) and Gram-negative bacteria (*X. campestris* pv. *holcicola*, *X. oryzae* pv. *oryzae*, *P. syringae* pv. *tomato* DC3000, *R. solanacearum*, and *E. coli* BL21) at a certain bacterial concentration (∼1 × 10^6^ CFU/ml). The bacteria plates treated with SM-985 (treatment plates) did not show any visible growth with SM-985 peptide at concentrations of 128, 64, 32, 16, 8, 4, and 2 μM. In contrast, the plates of the control showed growing colonies in all bacterial indicators. Thus, ≤2, considered the minimal SM-985 concentration, caused no visible colonies (complete death) against all bacterial indicators (Figure 2). The colonies in the plates of the control were counted for all bacterial indicators (Table 2).

**TABLE 2:** MLC of SM-985 peptide (CFU count).

Tested bacterial indicator	Control 10^5^ CFU/ml	SM-985 10^5^ CFU/ml
*C. fangii*	10.06 ± 1.51	–
*C. michiganesis* ssp. *michiganesis*	13.87 ± 0.13	–
*B. subtilis* 168	12.51 ± 2.50	–
*X. campestris* pv. *holcicola*	12.98 ± 1.27	–
*X. oryzae* pv. *orezae*	16.33 ± 3.60	–
*P. syringae* pv. *tomato* DC3000	17 ± 2.25	–
*R. solanacearum*	16.68 ± 2.69	–
*E. coli* BL21	16.16 ± 3.12	–

*The CFU/ml average of each bacterial indicator with standard deviation was counted. The SM-985 contraction was 2 μM, and the bacterial concentration was *∼* 1 *×* 10^6^ CFU/ml. The control was treated with dd water.*

#### SM-985 Increases Cell Membrane Permeability

**FIGURE 3:**
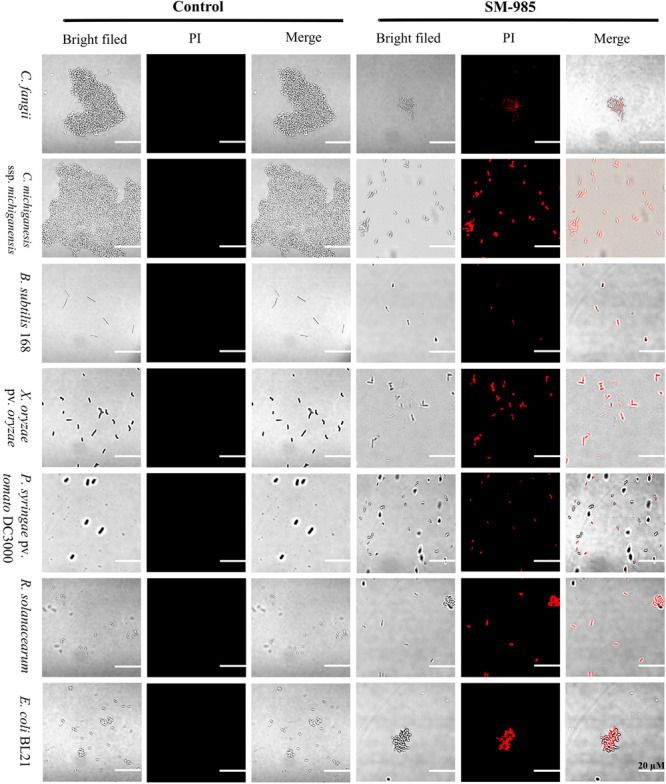
Visualizing the bacterial membrane permeability. The bacterial indicator, membranes (Gram-positive and Gram-negative) at a concentration of ∼1 × 10^7^ CFU/ml, lost integrity after treatment with 10 μM SM-985 for 4 h. However, the control did not take up the PI stain. No fluorescence indicates invariable membrane integrity. The red fluorescence of the PI stain indicates membrane disintegration. An Olympus BX61 laser scanning confocal microscope was used to observe PI uptake. Scale bar: 20 μm.

**FIGURE 4:**
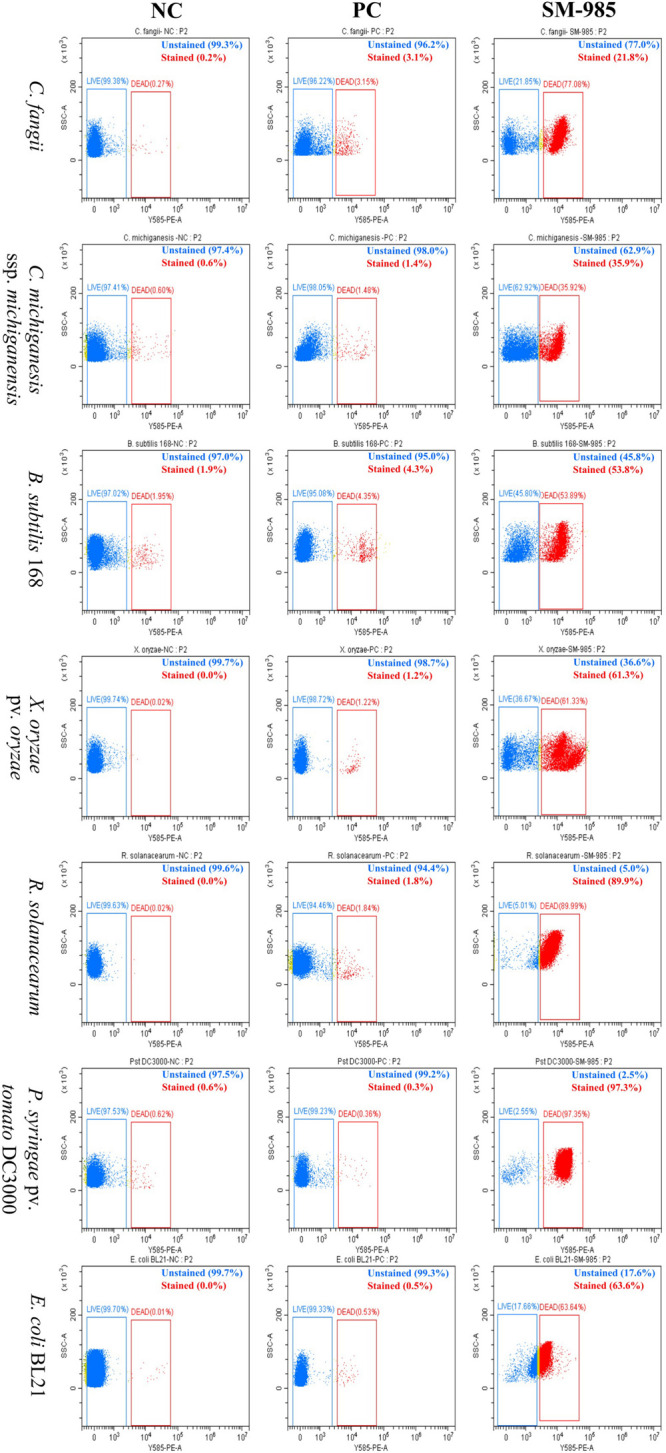
Quantitative analysis of bacterial membrane permeability. The PI uptake of the bacterial indicators (Gram-positive and Gram-negative) greatly increased after they were treated with 10 μM SM-985 for 4 h. The blue dots indicate unstained cells, while the red dots indicate stained cells. NC refers to the negative control and PC refers to the positive control. The Cytoflex lx machine was used to measure the PI uptake, and the data were analyzed with CyExpret 2.4 software.

PI dye uptake, which only can enter damaged cells, determines cell membrane damage. This experiment was performed on Gram-positive bacteria (*C. fangii*, *C. michiganesis* ssp. *michiganesis*, and *B. subtilis* 168) and Gram-negative bacteria (*X. oryzae* pv. *oryzae*, *P. syringae* pv. *tomato* DC3000, *R. solanacearum*, and *E. coli* BL21). An Olympus BX61 laser scanning confocal microscope was used to visualize PI uptake. Both cells treated with 10 μM SM-985 and controls were treated with PI dye. As a result, the bacterial cells treated with SM-985 were stained with PI and displayed red fluorescence of the PI dye, while the control bacterial cells showed no staining (Figure 3). The damage to the cell membrane was measured by Cytoflex lx (Beckman Coulter, Brea, CA, United States). There were two controls, the negative control representing bacterial cells without either SM-985 or PI dye, and the positive control representing bacterial cells without SM-985 and with PI dye. In this experiment, SM-985 disrupted the bacterial cell membrane, increasing the percentage of PI uptake more than both the negative and positive controls (Figure 4).

#### FITC-Labeled SM-985 Peptide Interacts With the Bacterial Cell Membrane

**FIGURE 5:**
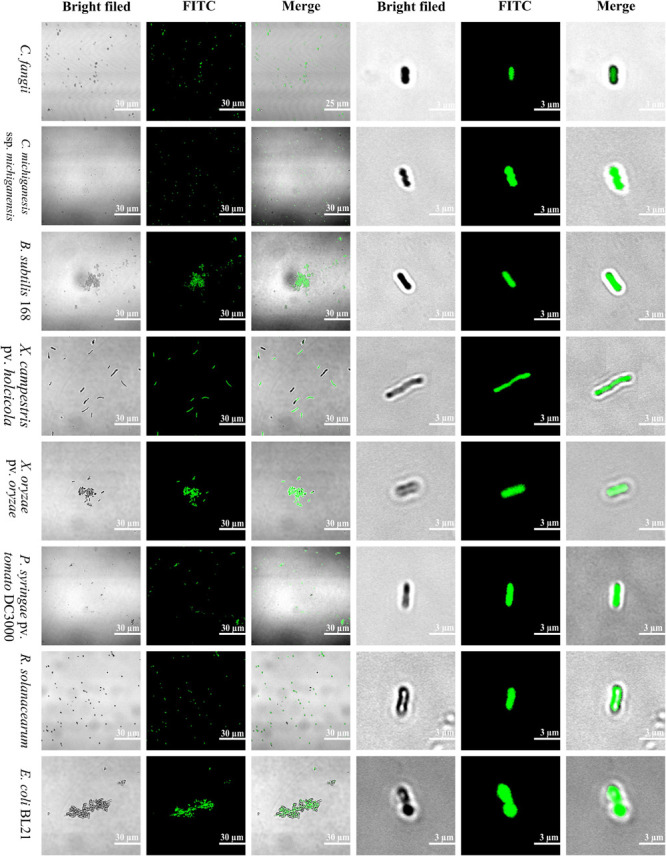
Localization of FITC-labeled SM-985. SM-985 showed a high affinity to bind to bacterial indicator (Gram-positive and Gram-negative) membranes after they were treated with 4 μM FITC-SM-985 for 4 h. The green fluorescence of the FITC tag indicates the interaction between SM-985 and bacterial membrane. The results were observed using an Olympus BX61 laser scanning confocal microscope. Scale bar: 30 and 3 μm.

**FIGURE 6:**
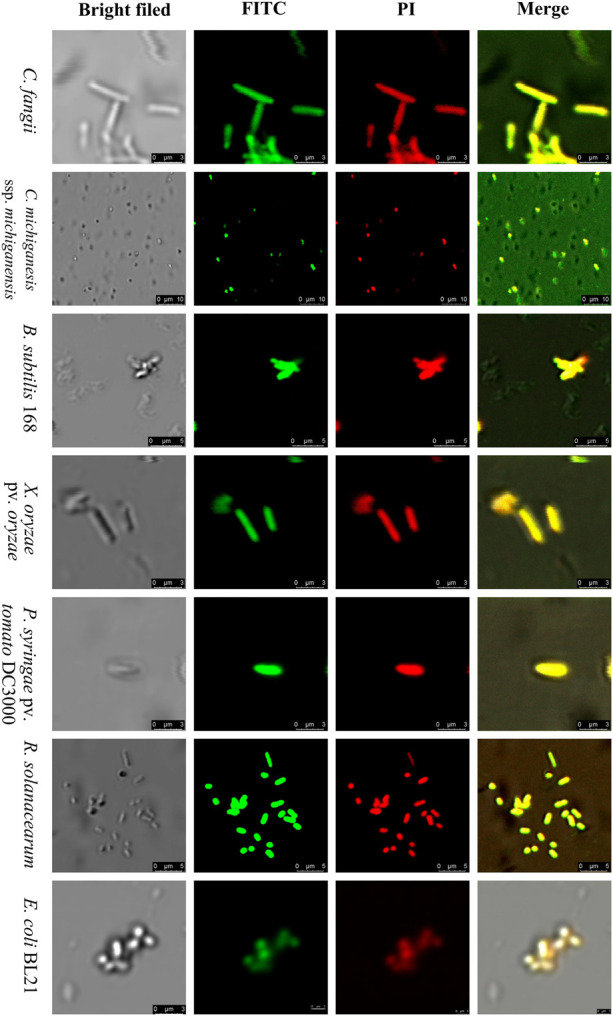
Bacterial membrane permeability by FITC-SM-985. The bacterial indicator membranes (Gram-positive and Gram-negative) lost their integrity after they were treated with 10 μM FITC-SM-985 for 4 h. The red fluorescence of the PI stain indicates membrane disintegration. The green fluorescence of the FITC tag indicates the interaction between SM-985 and bacterial membrane. The results were observed using a Leica TCS SP5 confocal microscope. Scale bar: 3, 5, and 10 μm.

FITC-labeled SM-985 peptide was used to explore the SM-985 working mechanism as AMPs, and the bacterial cells were treated with FITC-labeled peptide at a low concentration for 3 h. Both Gram-positive bacteria (*C. fangii*, *C. michiganesis* ssp. *michiganesis*, and *B. subtilis* 168) and Gram-negative bacteria (*X. campestris* pv. *holcicola*, *X. oryzae* pv. *oryzae*, *P. syringae* pv. *tomato* DC3000, *R. solanacearum*, and *E. coli* BL21) showed green fluorescence under an Olympus BX61 laser scanning confocal microscope, according to the suitable laser wavelength of FITC green fluorescence (Figure 5). These results showed that FITC-SM-985 peptide interfered with the bacterial cell membrane. Two antimicrobial activity assays were conducted to investigate the impact of the FITC tag on SM-985 antimicrobial activity. A cell killing assay was performed on *C. michiganesis* ssp. *michiganesis* and *P. syringae* pv. *tomato* DC3000 as representatives of other indicators. Both bacterial indicator suspensions (∼1 × 10^6^ CFU/ml) were treated with 5 μM FITC-SM-985 peptide for 4 h. However, the plates of the treated bacteria with FITC-SM-985 (treatment plates) did not show any visible growth, while the control plates showed growing colonies in both bacterial indicators (Table 3). Moreover, cell membrane integrity assay was conducted using PI uptake. Gram-positive bacteria (*C. fangii*, *C. michiganesis* ssp. *michiganesis*, and *B. subtilis* 168) and Gram-negative bacteria (*X. oryzae* pv. *oryzae*, *P. syringae* pv. *tomato* DC3000, *R. solanacearum*, and *E. coli* BL21) were stained with PI after treating each bacterial suspension (∼1 × 10^7^ CFU/ml) with 10 μM FITC-SM985 peptide. Under a confocal microscope, the bacterial cells showed both green fluorescence of FITC and red fluorescence of PI dye (Figure 6).

**TABLE 3:** Cell killing assay of FITC-SM-985 peptide (CFU count).

Bacterial indicator	Control 10^5^ CFU/ml	SM-985 10^5^ CFU/ml
*C. michiganesis* ssp. *michiganesis*	13.69 ± 1.66	–
*P. syringae* pv. *tomato* DC3000	14.70 ± 2.22	–

*The CFU/ml average of each bacterial indicator with standard deviation was counted. The SM-985 contraction was 5 μM, and the bacterial concentration was ∼1 × 10^6^ CFU/ml. The control was treated with dd water.*

#### *In vivo* Antimicrobial Activity of SM-985

**FIGURE 7:**
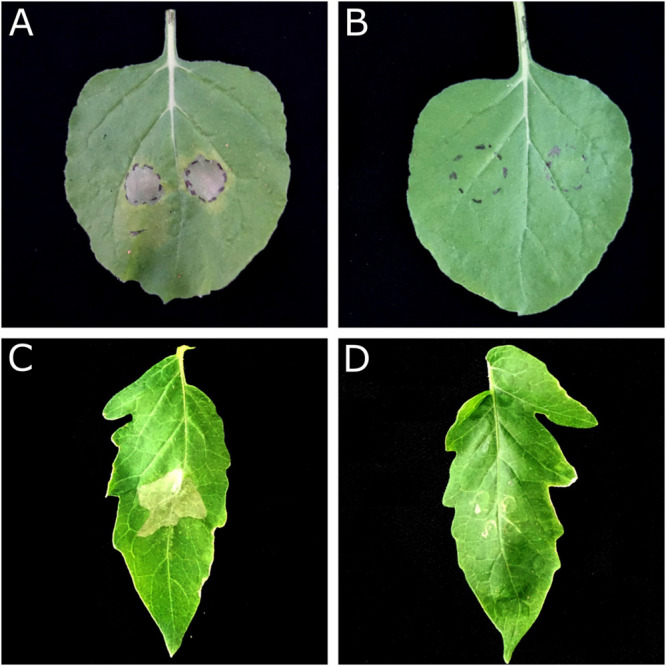
*In vivo* antimicrobial activity assay by the leaf infiltration method. Pst DC3000 at a concentration of ∼1 × 10^6^ CFU/ml lost its ability to cause HR on *N. benthamiana* and necrosis on *S. lycopersicum* after treatment with 5 μM SM-985 for 4 h. **(A)** *N. benthamiana* treated with dd water (control). **(B)** *N. benthamiana* treated with SM-985. **(C)** *S. lycopersicum* treated with dd water (control). **(D)** *S. lycopersicum* treated with SM-985. The results were observed after 48 h for *N. benthamiana* and 96 h for *S. lycopersicum*.

**FIGURE 8:**
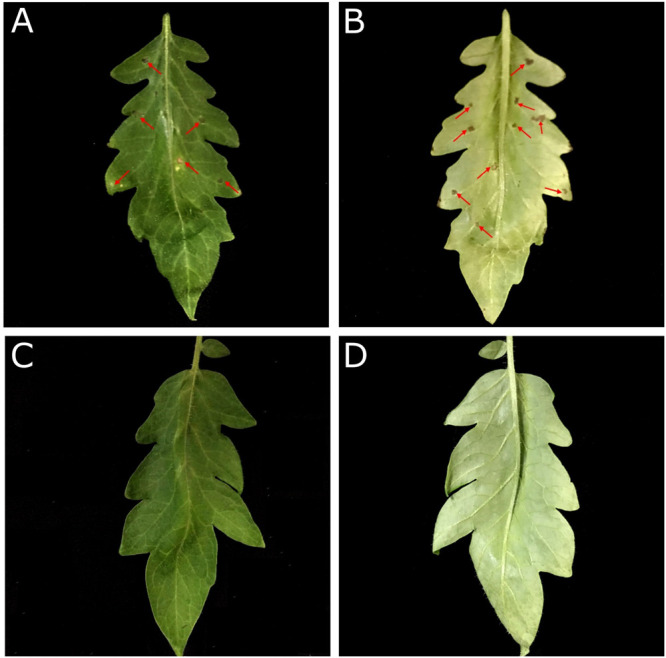
SM-985 prevented leaf spot infection on tomato. Pst DC3000 at a concentration of ∼1 × 10^6^CFU/ml was treated with 5 μM SM-985, while the control was treated with dd water. Both the SM-985 treatment and control were directly sprayed on *S. lycopersicum* (adaxial and abaxial surfaces), and the results were observed after 6 days. **(A)** Control adaxial surface. **(B)** Control abaxial surface. **(C)** SM-985 adaxial surface. **(D)** SM-985 abaxial surface. The red arrows indicate leaf spots.

Based on the ability of *P. syringae* pv. *tomato* DC3000 to cause leaf spot disease on *S. lycopersicum* and the hypersensitivity reaction (HR) on *N. betnhamiana*, the Pst DC3000 bacterial suspension (∼1 × 10^6^ CFU/ml) was treated with 5 μM SM-985 for 4 h while the control was treated with dd water. Both hosts were inoculated with the treated and control bacterial cells using the infiltration technique. The treated Pst DC3000 bacterial cells could not induce disease symptoms on either *S. lycopersicum* or HR on *N. betnhamiana.* In contrast, the control Pst DC3000 bacterial cells induced disease symptoms on *S. lycopersicum* and HR on *N. betnhamiana* after 4 and 2 days, respectively (Figure 7). To simulate the real infection conditions of leaf spot disease, SM-985 was added to the Pst DC3000 bacterial suspension (∼1 × 10^6^ CFU/ml) at a final concentration of 5 μM by direct spraying on the *S. lycopersicum* leaves. The Pst DC3000 treated with SM-985 did not show symptoms, while the control Pst DC3000 infected the *S. lycopersicum* leaves on both adaxial and abaxial surfaces and caused leaf spot symptoms (Figure 8).

#### SM-985 Causes Distinct Damage to the Bacterial Cell Membrane

**FIGURE 9:**
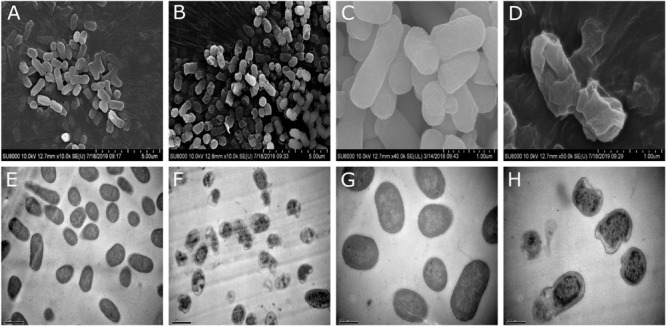
Cell membrane damage investigation by SEM and TEM. A bacterial suspension of ∼1 × 10^7^ CFU/ml of *C. michiganesis* ssp. *michiganesis* was treated with 15 μM SM-9895 for 4 h. **(A,C,E,G)** The cytoplasmic membranes were intact, and the cells appeared normal in the control. **(B,D,F,H)** SM-985 caused damage to the cytoplasmic membrane and cell lysis. The results observed using a HITACHI SU8010 scanning electron microscope and HITACHI H-7650 transmission electron microscope.

From previous results, it was determined that SM-985 damaged the cell membrane by increasing cell membrane permeability. SEM and TEM assays were performed on *C. michiganesis* ssp. *michiganesis* to observe the cell membrane damage after treating the bacteria cells with SM-985. The bacterial suspension (∼1 × 10^7^ CFU/ml) was treated with 15 μM SM-985 for 4 h, while the control was treated with dd water. The SEM images showed the damaged and disrupted cell envelope of the treated *C. michiganesis* ssp. *michiganesis*. In contrast, the cell envelope of the control cells was intact and had a regular shape (Figure 9). Moreover, the TEM images revealed cell lysis, a damaged cell membrane, and the absence of cytoplasmic material in the treated *C. michiganesis* ssp. *michiganesis*. The cell membrane of the control cells was intact and smooth, and the cells were full of cytoplasmic material (Figure 9).

#### Calcium Chloride Inhibits SM-985 Antimicrobial Activity

**FIGURE 10:**
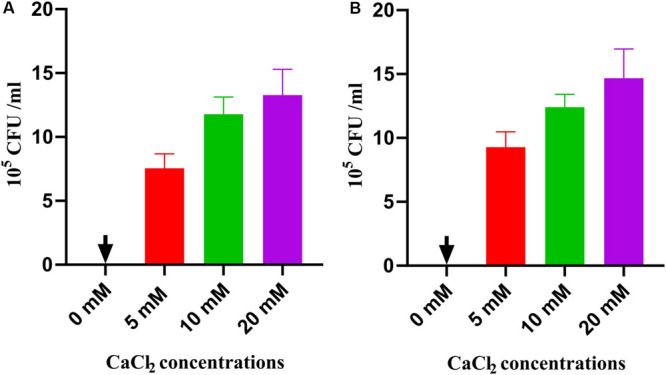
SM-985 sensitivity to CaCl_2_ salt. Bacterial suspension (CFU/ml) treated with 5 μM SM-985 for 4 h. CaCl_2_ salt was added at different concentrations. SM-985 antimicrobial activity was noticeably decreased after CaCl_2_ addition. The CaCl_2_ salt and SM-985 had an inverse relationship **(A)** *C. michiganesis* ssp. *michiganesis*. **(B)** Pst DC3000. Results with standard deviation are shown.

SM-985 peptide activity was affected by adding calcium chloride salt. The results of both the Gram-positive bacterial indicator *C. michiganesis* ssp. *michiganesis* and Gram-negative bacterial indicator *P. syringae* pv. *tomato* DC3000 showed that in 0 mM calcium chloride, SM-985 activity was very strong, with no visible colony growth. However, after addition of 5 mM calcium chloride, SM-985 activity was noticeably decreased. The salt concentration and SM-985 activity showed an inverse relationship. Thus, 10 mM calcium chloride caused more growing colonies than 5, and 20 mM caused more growing colonies than 10 mM. In summary, increasing the calcium chloride concentration resulted in more growing colonies and reduced the SM-985 antimicrobial activity (Figure 10).

## Discussion

High losses caused every year by plant pathogens in addition to the restriction of the use of pesticides in several countries around the world have urged the development of alternative approaches as new antimicrobial agents (Giralt, 2014). This study focused on isolating novel AMPs with strong antimicrobial activity against several bacterial plant pathogens. A wild plant species, *Z. mays* ssp. *mexicana*, was chosen as the source of the AMPs. Teosinte is the wild ancestor of maize (Doebley, 1992). Some genes, such as resistance genes, may be lost during the domestication of plant species (Chaudhary, 2013), or the expression of certain genes linked to the resistance of plant diseases may be expressed more in wild plants such as teosinte than domesticated plants such as maize (Dávila-Flores et al., 2013; Szczepaniec et al., 2013). Therefore, *Z. mays* ssp. *mexicana* is a good candidate source for the isolation of AMPs.

Toward this aim, a cDNA library of teosinte was constructed. After sequencing, it was confirmed that *Z. mays* ssp. *mexicana* was the source of the cloned insertions via BLAST analysis. Based on the cDNA sequences, a translation tool was used to convert them to amino acid sequences. *In silico* predictive tools may be beneficial for big-scale screening and evaluation of new AMPs. Therefore, five servers were used to screen the amino acid sequences for potent AMPs. These servers utilize several algorithms to predict new AMPs (Liu et al., 2017), and this prediction is based on different parameters. More than one server was used to increase the prediction accuracy. According to the prediction results, SM-985 peptide showed the highest prediction values among the other amino acid sequences. SM-985 is a short peptide consisting of 21 amino acids and is similar to some well-known AMPs with a size range between 10 and 50 amino acids (Hamamoto et al., 2002). The short and simple sequence of an AMP may simplify its rapid construction, cut costs of synthesis, and accelerate translational applications (Ong et al., 2014). SM-985 is composed of eight different amino acids: arginine (R) 38%, glycine (G) 19%, tryptophan (T) 14%, proline (P) 9%, alanine (A) 4%, isoleucine (4%), histidine (4%), and threonine (4%). As a result, the APD3 database considered SM-985 as arginine-rich peptide due to its high content of arginine. Some studies have demonstrated that arginine is the key structure of cell-penetrating peptides (CPPs) (Takeuchi and Futaki, 2016; Murayama et al., 2017). Previous studies have shown that arginine-rich peptides appear to be closer to the lipid bilayer in simulations, validating their increased ability to initiate probable translocation events compared with variations of other peptides (Takeuchi and Futaki, 2016). In addition to arginine, SM-985 is composed of other amino acids, also all of which have potential antimicrobial activity. Several studies have mentioned the antimicrobial potential of tryptophan (Courrol et al., 2019), glycine (Halder et al., 2019), and proline (Mardirossian et al., 2019). SM-985 is a CAMP due to the high content of arginine (Zhang and Gallo, 2016).

SM-985 was predicted as an α-helical peptide with an α-helix secondary structure, and the α-helical conformation has been directly linked to the antimicrobial activity (Park et al., 2000). In previous studies, many AMPs with an α-helix structure have been reported (Bonduelle, 2018; Shen et al., 2018; Triana-Vidal et al., 2018) due to the helical structure, which plays an essential role in AMP activity, whereas the α-helix secondary structure assumes an amphipathic structure and presents a precise hydrophobic portion for membrane penetration (Zelezetsky and Tossi, 2006). The 3D structural model of SM-985 was predicted using the I-TASSER server, and this model was validated with ProSA-web and MolProbity software. The ProSA-web results showed that the SM-985 3D model was within the favorable region of structures, and these results were comparable to a previous study (Liscano et al., 2019). The results of MolProbity indicated that the peptide structure parameters remained within the limits of acceptable quality and stability (Beg et al., 2018).

Due to the high levels of positive amino acids such as arginine, the net charge of SM-985 is (+8). Most CAMPs have such a positive net charge, ranging from +4 to +8, which are ideal for biotic operations (Tossi et al., 2000). CAMPs bind to cytoplasmic membrane phospholipids by forming a forceful electrostatic link (Yount et al., 2006). In a previous study, the net charge of V13K analogs was decreased to less than +4, which inactivated the peptide, while higher antimicrobial activity was observed when they increased the net charge from +4 to +8. However, a further rise in net charges to +9 and +10 increased the toxicity of the peptide (Jiang et al., 2008).

Hydrophobicity is another important feature of AMP activity. Creating pores in the cytoplasmic membrane of the bacterial cell requires hydrophobic residues that interact with the lipid bilayer (the hydrophobic portion). Ultimately, this interaction results in the degradation of the bacterial cytoplasmic membrane. Low hydrophobicity may be insufficient to induce an effective interaction between the AMPs and cytoplasmic membranes, which decreases antimicrobial activity. Nevertheless, the exceptionally high hydrophobicity of AMPs, which leads to their auto-association, makes them unable to pass through the cell wall of bacteria. The hydrophobic ratio of SM-985 is 23%, which represents moderate hydrophobicity; moreover, the average hydrophobicity is vital for optimal antimicrobial activity (Chen et al., 2007). The helical wheel of SM-985 indicated amphipathic α-helix conformations, and an earlier study has shown that the positive polar face supports phospholipid binding when the AMP is attached to the cell membrane. At that point, via hydrophobic interactions, the non-polar face of the AMP inserts into the membrane (He et al., 2018). The Boman index of SM-985 is 5.19 kcal/mol, and the high positive Boman index increases the ability of SM-985 to bind to bacterial cell membrane proteins (He et al., 2018). Radzicka and Wolfenden established an early hydrophobic scale focused on the partitioning of small-molecule side-chain amino acid analogs between water and cyclohexane. The Boman index is essentially the average hydrophobic value measured using the Radzicka–Wolfenden scale (Radzicka and Wolfenden, 1988).

According to the BLAST results, SM-985 showed no significant similarity with other AMPs from three large AMPs databases, which strongly indicates that it is a new plant AMP from teosinte. The prediction results and physiochemical properties of SM-985 make it a promising AMP candidate. In our studies, we also confirmed the *in silico* results experimentally.

A critical function of AMPs is the direct killing of microbial targets. In our research, MICs and MBCs of SM-985 have been measured against a wide range of bacterial indicators, including Gram-positive bacteria (*C. fangii*, *C. michiganesis* ssp. *michiganesis*, and *B. subtilis* 168) and Gram-negative bacterial indicators (*X. campestris* pv. *holcicola*, *X. oryzae* pv. *oryzae*, *P. syringae* pv. *tomato* DC3000, *R. solanacearum*, and *E. coli* BL21). Interestingly, the MICs and MBCs values of SM-985 were higher against Gram-positive bacterial indicators than Gram-negative bacterial indicators. The MICs of SM-985 against Gram-positive and most Gram-negative indicators were 8 and 4 μM, respectively. Thus, Gram-positive bacterial indicators are more tolerant to SM-985 than Gram-negative bacterial indicators because of the cell envelop structure. There are more layers of peptidoglycan surrounding Gram-positive membranes than Gram-negative membranes (Silhavy et al., 2010), which might explain the difference in MIC and MBC values. It is interesting to note that Guavanin 2 peptide (Porto et al., 2018) shares common features with SM-985, both of which are arginine-rich α-helical peptides with no similar sequences in AMPs databases, and have a preference for Gram-negative bacteria. Moreover, a previous study examined NCR335 antimicrobial activity against two bacterial indicators. The MIC of CNR335 against *Listeria monocytogenes* (Gram-positive bacteria) was 32 μM, while the MIC of NCR335 against *Salmonella enterica* (Gram-negative bacteria) was 16 μM (Farkas et al., 2017). The next step in our research was to investigate the lowest concentration value of SM-985 that triggered direct killing of both Gram-positive and Gram-negative bacterial indicators using 10 mM phosphate buffer. The MLC of SM-985 was ≤2 μM, and we noticed that the MLC value was lower than the MIC and MBC values. Thus, SM-985 demonstrated lower antimicrobial efficacy with MIC and MBC agar dilution compared with the MLC determination process. The main reason for the observed difference was the nature of the place in which the antimicrobial activity was investigated. The agar dilution process uses MHB medium, which contains high divalent cation levels, whereas the 10 mM phosphate buffer used in the MLC system does not contain divalent cations. Divalent cation inhibition is considered to be a common feature of CAMPs (Farkas et al., 2017).

SM-985 increased the bacterial indicator membrane permeability, which was confirmed by PI uptake assay. Only dead bacterial cells with damaged cell membranes can take up PI dye, and both Gram-negative and Gram-positive indicators treated with SM985 were able to take up more PI dye than the control. Many studies have confirmed the cell membrane damage after treatment with AMPs by the PI uptake assay (Farkas et al., 2017; Velivelli et al., 2018; Yang M. et al., 2018). The positive net charge of SM-985 facilitates the interaction with the cell membrane phospholipid negatively charged groups (Fernandez et al., 2013). Therefore, SM-985 interacts with bacterial cell membranes to induce damage. As a result, the bacterial cell is able to take up the PI dye. The interaction between SM-985 and the bacterial cell membrane was confirmed by treating the bacterial indicators with FITC-SM-985, and as a result, SM-985 showed a strong affinity to the bacterial membrane (both Gram-positive and Gram-negative). These results are consistent with other studies on CAMPs (Farkas et al., 2014; Zhu et al., 2015b; Nagarajan et al., 2019). The effect of the FITC tag on SM-985 antimicrobial activity was investigated by performing antimicrobial activity assays. In the cell killing assay, FITC-SM-985 was able to kill Gram-positive *C. michiganesis* ssp. *michiganesis* and Gram-negative *P. syringae* pv. *tomato* DC3000. Moreover, in the PI uptake assay, PI dye uptake ability of all the bacterial indicators was increased after treating them with FITC-SM-985, and these results were agreement with a previous study (Van De Velde et al., 2010). From recent findings, even for the short amino acid sequence of SM-985, the FITC tag did not affect SM-985 antimicrobial activity.

Pst Dc3000 was treated with SM-985 at a concentration of 5 μM and then incubated for 4 h at 28°C. Then, *N. betnhamiana* and *S. lycopersicum* were inoculated with Pst DC3000 using the infiltration method. SM-985 prevented Pst DC3000 infection on *N. betnhamiana* and *S. lycopersicum*. The infection ability of Pst DC3000 was lost due to the incubation period of Pst DC3000 with SM-985. To simulate the real infection conditions, Pst DC3000 was treated with SM-985 at a concentration of 5 μM and then directly sprayed on *S. lycopersicum* without an incubation period. Pst DC3000 lost infection ability after it was treated with SM-985. It is known that leaf spot disease pathogens require a prolonged period (12–24 h) to initiate infection, and during this period, SM-985 interacts with the PsDC3000 membrane and damages it, leading to cell death. The *in vivo* antimicrobial activity of SM-985 opens the door for the application of SM-985 to protect plants against bacterial pathogens.

Scanning electron microscopy and TEM demonstrated the damaging effect of SM-985 on the bacterial membrane. The SEM images showed the damaged and disrupted cell envelope of the *C. michiganesis* ssp. *michiganesis* after it was treated with 15 μM SM-985 for 4 h. In contrast, the cell envelope of the control cells appeared intact with a regular shape. The TEM images demonstrated cell membrane lysis and the lack of cytoplasmic material in the treated *C. michiganesis* ssp. *michiganesis*. In contrast, the cell membrane of the control cells was intact and smooth, and the cells were full of cytoplasmic material. These results are comparable to many other studies used SEM and TEM to determine the damage present in the bacterial cell membrane after treatment with AMP (Wu et al., 2014; Zhang et al., 2018; Halder et al., 2019; Hou et al., 2019).

Calcium chloride impairs the antimicrobial activity of SM-985 on *C. michiganesis* ssp. *michiganesis* (Gram-positive bacteria) and *P. syringae* pv. *tomato* DC3000 (Gram-negative bacteria), and SM-985 shows sensitivity to different concentrations of calcium chloride. In fact, several studies have suggested a detrimental effect of divalent cations (Ca^+2^) on the antimicrobial activity of AMPs (Maisetta et al., 2008; Huang et al., 2011). The mechanism of SM-985 calcium chloride sensitively might be clarified as follows: the positive charge of calcium chloride disorganizes the interaction between the cationic SM-985 and the bacterial membrane (Zhu et al., 2015a). Other studies have indicated that salt sensitivity might affect AMP stability, which affects antimicrobial activity (Luo et al., 2018).

## Conclusion

We screened a cDNA library from teosinte (*Z. mays* ssp. *mexicana*) for AMPs using *in silico* prediction tools, and SM-985 peptide was predicted as an AMP. SM-985 showed very promising physiochemical properties as an AMP with an arginine-rich α-helical structure. SM-985 showed wide spectrum antimicrobial activity against bacterial phytopathogens; Gram-positive and Gram-negative plant pathogenic bacteria were efficiently eliminated by cationic plant peptide SM-985. Thus, SM-985 was demonstrated to disrupt and damage the bacterial cell physical structure by increasing the membrane permeability through pore formation. *In vivo* antimicrobial activity was conducted, and SM-985 prevented leaf spot infection caused by Pst DC3000 on *S. lycopersicum*. In addition, SM-985 demonstrated sensitivity to calcium chloride acid, a typical characteristic of CAMPs.

## Data Availability Statement

The raw data supporting the conclusions of this article will be made available by the authors, without undue reservation, to any qualified researcher.

## Author Contributions

AQ and WD conceived research and designed the experiments. AQ conducted library construction and data analyses. AQ and FW performed gene screening and functional test experiments. AQ and WD wrote the manuscript with input from all co-authors.

## Conflict of Interest

The authors declare that the research was conducted in the absence of any commercial or financial relationships that could be construed as a potential conflict of interest.

## Footnotes

1 https://blast.ncbi.nlm.nih.gov/Blast.cgi

2 http://mmgdb.hzau.edu.cn/

3 https://www.maizegdb.org

4 https://web.expasy.org

5 http://www.camp.bicnirrh.res.in/

6 http://aps.unmc.edu/AP/main.php

7 http://tcoffee.crg.cat/apps/ampa/do:ampa

8 https://dbaasp.org/prediction

9 http://biotechlab.fudan.edu.cn/database/lamp/

10 http://www.bicnirrh.res.in/classamp/predict.php

11 http://cabgrid.res.in:8080/amppred/server.php

12 https://webs.iiitd.edu.in/raghava/antibp/index.html

13 http://csb.cse.yzu.edu.tw/dbAMP/

14 http://bioinf.cs.ucl.ac.uk/psipred/

15 https://zhanglab.ccmb.med.umich.edu/

16 http://osddlinux.osdd.net/raghava/pepstrmod/index.php
