# Corrigendum: Prediction and Characterization of Cationic Arginine-Rich Plant Antimicrobial Peptide SM-985 From Teosinte (*Zea mays* ssp. *mexicana*)

**DOI:** 10.3389/fmicb.2021.667085

**Published:** 2021-03-03

**Authors:** Abdelrahman M. Qutb, Feng Wei, Wubei Dong

**Affiliations:** ^1^Department of Plant Pathology, College of Plant Science and Technology and the Key Lab of Crop Disease Monitoring and Safety Control in Hubei Province, Huazhong Agricultural University, Wuhan, China; ^2^Department of Agricultural Botany, Faculty of Agriculture, Al-Azhar University, Cairo, Egypt; ^3^State Key Laboratory of Agricultural Microbiology, College of Life Science and Technology, Huazhong Agricultural University, Wuhan, China

**Keywords:** antimicrobial peptide, AMP prediction, bacterial pathogens, membrane damage, teosinte

In the published article, there was an error regarding the affiliations for Abdelrahman M. Qutb. As well as being affiliated with the Department of Plant Pathology, College of Plant Science and Technology and the Key Lab of Crop Disease Monitoring and Safety Control in Hubei Province, Huazhong Agricultural University, Wuhan, China, they should also have Department of Agricultural Botany, Faculty of Agriculture, Al-Azhar University, Cairo, Egypt.

The authors apologize for this error and state that this does not change the scientific conclusions of the article in any way. The original article has been updated.

