# Supplementary material for: Prediction and Characterization of Cationic Arginine-Rich Plant Antimicrobial Peptide SM-985 From Teosinte (Zea mays ssp. mexicana)
Source: Front Microbiol. 2020 Jun 19;11:1353. doi: 10.3389/fmicb.2020.01353 (PMC7318549; doi:10.3389/fmicb.2020.01353)
Supplement: Supplementary file 1 [file Data_Sheet_1.PDF]

Supplementary Materials

Prediction and characterization of cationic arginine-rich plant antimicrobial peptide SM-985 from Teosinte (*Zea may* ssp. *mexicana*)

Abdelrahman M Qutb, Feng Wei, Wubei Dong

Construction of the cDNA Library

A mixed sample was created from 10 timepoints samples. Total RNA was extracted from the mixed sample according to the TRIzol reagent protocol. The mRNA was isolated using the PolyATtract® mRNA Isolation System kit (Part# TM021) from Promega®. The double-stranded cDNA was synthesized from polyA+ mRNA using the PrimeScript ™ Double Strand cDNA Synthesis Kit taKaRa® with specific oligo dT primers (contains *Xba* I cleavage site). Three pairs of adaptors containing the cleavage site of *Nde* I were added to the cDNAs **(Table S1)**. cDNAs and cloning vector pBE-S DNA were digested with *Nde* I and *Xba* I restriction endonucleases enzymes. The linear pBE-S DNA and cDNAs were purified after digestion using a gel recovery kit and clean-up kit, respectively. cDNA fragments were ligated with the vector using T_4 DNA ligase. The ligation product was first transformed into *E. coli* HST08 competent cells for propagation. After transformation, the *E. coli* cells were cultured overnight on LB plates with ampicillin (100 µg/ml). Plasmids were extracted from the growing transformant colonies using the EasyPure® Plasmid MiniPrep Kit. The extracted plasmids were transformed into *B. subtilis* SCK6 supercompetent cells to enhance the transformation efficiency. After transformation, the *B. subtilis* cells were cultured on LB plates with kanamycin (10 µg/ml). The positive recombinant clones were collected, grown in liquid LB medium with kanamycin and then stored in a -80 ℃ freezer.

Preparation of 10 mM Sodium Phosphate Buffer

To create 1 L of 10 mM sodium phosphate buffer (Works 2018), stock solution A (20 mM sodium phosphate dibasic dihydrate $Na_2HPO_4.2H_2O$) was prepared by 3.56 g/L dissolving

$Na_2HPO_4.2H_2O$. Solution B (20 mM sodium phosphate, monobasic, monohydrate $NaH_2PO_4•H_2O$) was generated by dissolving 2.76 g/L $NaH_2PO_4•H_2O$. To make 10 mM phosphate buffer pH 7.0, 350 ml of solution A and 195 ml of solution B were mixed, and the volume was raised to 1 L with dd water. The buffer was then sterilized by autoclaving for 20 min.

Preparation of the Bacterial Suspension:

Preparation of the Bacterial Suspension

The bacterial indicators were grown to an OD600 of 0.3, and then the pellet of 1 ml of bacterial culture was washed two times with 10 mM sodium phosphate buffer (pH 7.0) and centrifugation at 6000 rpm for 2 min. Next, the pellet was resuspended and diluted in the buffer until the OD600 reached 0.1. A hemocytometer and light microscope were used to calculate the CFU/ml of each bacterial indicator suspension with OD600 0.1. To reach ~1 x 10^6 CFU CFU/ml, a relative volume of each bacterial suspension with an OD600 of 0.1 containing ~1 x 10^6 CFU was diluted in 1 ml of buffer **(Table S6).**

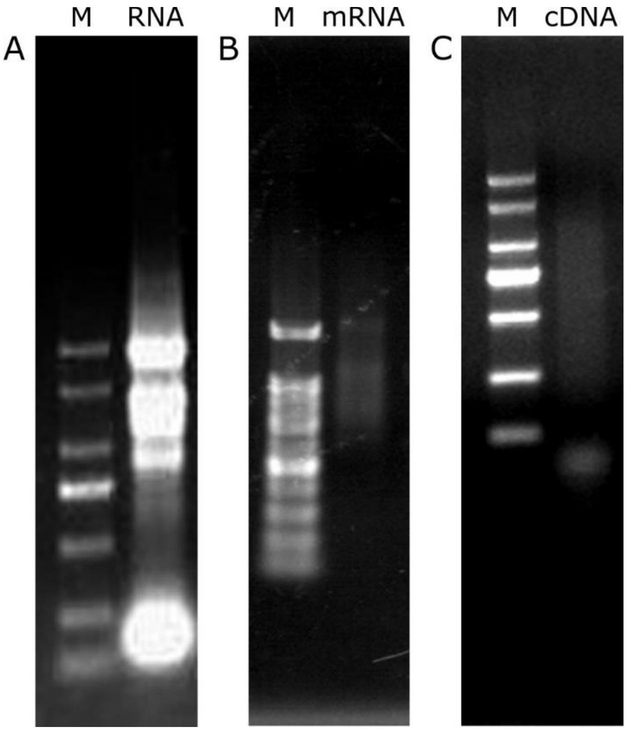

Figure S1. Quality of the total RNA, mRNA, and cDNA. (A) Total RNA and marker and (B) mRNA. (C) cDNA. The marker for (A) and (C) is DL2000, while the marker for (B) is 100 bp.

.

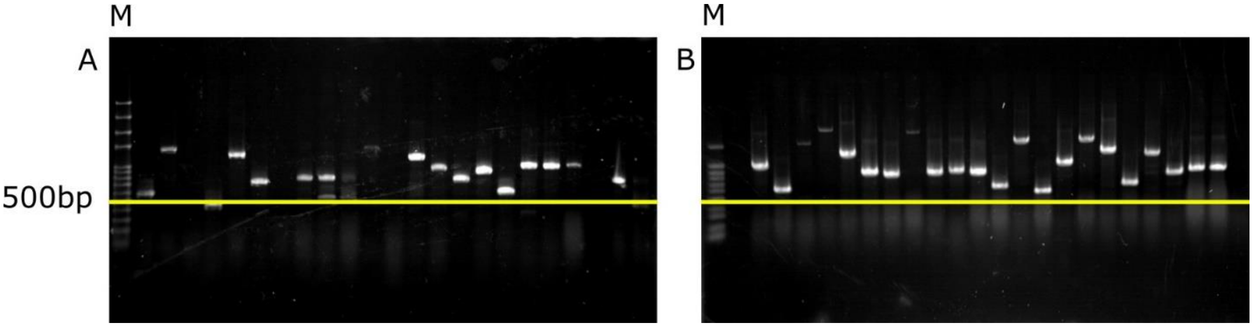

Figure S2. Random insert sizes in the teosinte cDNA library. (A) After transformation into *E. coli* HST08. (B) After transformation into *B. subtilis* SCK. The yellow line represents the empty vector band size (500 bp). The marker in (A) and (B) is 100 bp.

Table S1. Sequences of Adaptors

Adaptor	Sequence
1	CTCGAGAGGAATTC**CATATG**C
2	G**CATATG**GAATTCCTCTCGAGTACG
3	CTCGAGAGGAATTC**CATATG**CT
4	AG**CATATG**GAATTCCTCTCGAGTACG
5	CTCGAGAGGAATTC**CATATG**CTA
6	TAG**CATATG**GAATTCCTCTCGAGTACG

Table S2. General pBE-S DNA primers

**Forward primer**	GTTATTTCGAGTCTCTACGG
**Reverse primer**	TAACCAAGCCTATGCCTACA

Table S3. The prediction results of 30 peptides which showed prediction values above 0.5

Peptide name	Sequence	Prediction value
SS-168	TVIICIYIFLLLSGALVAAG	0.51
SM-223	IGTIPIQDLPVYEASAAAGAHMVELH	0.61
SM-288	SCFWYHVLPPSCIPFRGECICTPEGGQPLSHH IFKFLPGIVSVRNGPSTKIRRNGVVESPWRW CYLQRIS	0.62
SS-350	PFFCRTLPMLLPAVPASVSA	0.62
SM-433	AGVLTVAAAAMTAKMYATAAAGAHMHSE FF	0.64
SS-811	KEVLVKEVQRLQLALEEQTE	0.66
SM-901	IRNACLDITIITTNAVVYHSIANAVRHRVNLT	0.54
**SM-985**	**GAGIGPGHRRTWRRWPRRRWR**	**0.99**
SS-1050	RSSSSSAPSPWM	0.51
SL-1220	YINQQKKSFFNKKRIILSSIVVLFLIIGGAFLA AGAHMELGTLEGSEFKLVD	0.60
SL-1230	SSERLRNNRLYNLRLRCGGSLVTTHFVLRIT SEFLRVSCLNFPFAFFKTLLIICLLQYLNIHLA DDSLCC	0.59
SM-1293	HISALHTLRRAIVLRQSLSFIVIKG	0.77
SL-1378	MKKWLIIAVSLAIAIVLFMYTKGEAKAAAG AMLGRWIYSLVELEQGGQYLVPAVFSRRKI LELRLLVLSLLKVTYSPVENLVHIRSRELAQ DLFQGTWIAIFLMK	0.80
SL-1464	LSFGQPFFPSMILTVQAAKSAAGAHMKPSV	0.59

SM-1466	WDFVMPRLGRNTSKKKPCLLISTIRNVQVV	0.64
SM-1491	DGSKRLESPVKNDPVLVLLRSRMLSLGCCL	0.58
SS-1570	GPPREKCPSEIRRPY	0.69
SS-1577	CKIMGSRLINLQV	0.63
SM-1581	KSQFSAHFNRNRIHTNCRSSSGGRCTYAAV VYIS	0.55
SM-1588	KTKASIKFGICVGLLCLSITGFT	0.83
SM-1700	TIFAFLTGLMMFGTITAASAAAG	0.79
SM-1722	MFAKRFKTSLLPLFAGFLLLFH	0.57
SM-1770	ASAAAGAHMEQAGVSFCCATHC	0.79
SS-1777	FAKRFKTSLLPLFAGFL	0.57
SM-1801	HLVLAGPAAASAAAGAHMEQAGVSFCCAT HCL	0.72
SM-1830	HALDYIGAAAGAHKCSCDLVGRCGDLVRV ASWR	0.61
SM-1833	RAGNVLAAGGGPRARQRSSTRRSYTSPHICF LA	0.75
SM-1850	ELEGEWREDEGALLLQDLADVIAG	0.69
SM-1890	PLRAPCAGLGTIGGAASFYFTRPFGGV	0.66
SS-1999	DPALPSLGHVFTSGCFVKLF	0.54

After the translation of the 2000 cDNA inserts to amino acid sequences, each amino acid sequence was named based on size (the amino acid sequences were grouped under three groups small sequences 0-20 aa, medium sequences 21-50 aa, and large sequences above 50 aa) and the serial number of each sequence from 1 – 2000. The 2000 sequences were screened for AMPs by the CAMP$_{R3}$ prediction server (SVM algorithm) and based on the screening results, 30 sequences showed prediction values above 0.5 out of 1.0. SM-985 showed the heights prediction value among the 30 peptides.

TABLE S4. Antimicrobial peptide prediction results of SM-985

SM-985 sequence	GAGIGPGHRRTWRRWPRRRWR		
**Sever**	**Algorithms**	**Class (AMP/NAMP)**	**AMP probability**
**$CAMP_{R3}$**	SVM	AMP	0.99
	RF	AMP	0.86
	ANN	AMP	-
	DA	AMP	0.99
**DBAASP**	DBAASP	AMP	-
**MLAMP**	RF	AMP	0.60

SM-985 predicted as AMP by all the servers and algorithms. SVM, RF, ANN, and DA refer to the Support Vector Machine, Random Forest, Artificial Neural Network, and Discriminant Analysis, respectively. The AMP probability values varied among the servers, and all of them are out of 1; however, some did not provide a prediction value and represented in (-). NAMP refers to non-AMP.

TABLE S5. The classification of SM-985 prediction

Sever	Algorithm	Class	probability
**ClassAMP**	RF	Antibacterial	0.83
**Antibp**	ANN	Antibacterial	1.00
**MLAMP**	RF	Antibacterial	0.88
**dbAMP**	RF	Antibacterial	0.96

SM-985 predicted as antibacterial peptide by all the servers and algorithms. RF and ANN refer to Random Forest and Artificial Neural Network, respectively. The AMP probability values varied among the servers, and all of them are out of 1.

Table S6. The relative volume of bacterial suspension (OD600 0.1) containing ~1 x 10^6 CFU

Bacterial strain	Volume (µl)
C. fangii	17
C. michiganesis ssp. *michiganesis*	17
B. subtilis 168	40
X. campestris pv. *holcicola*	15
X. oryzae pv. *oryzicola*	15
P. syringae pv. *tomato* DC3000	22
R. solanacearum	13
E. coli BL21	20
